# Long-term, continuous infusion of single-agent dinutuximab beta for relapsed/refractory neuroblastoma: an open-label, single-arm, Phase 2 study

**DOI:** 10.1038/s41416-023-02457-x

**Published:** 2023-10-10

**Authors:** Holger N. Lode, Karoline Ehlert, Stephanie Huber, Sascha Troschke-Meurer, Nikolai Siebert, Maxi Zumpe, Hans Loibner, Ruth Ladenstein

**Affiliations:** 1 Department of Pediatric Hematology and Oncology, University Medicine Greifswald, Greifswald, Germany; 2 Anyxis Immuno Oncology, Vienna, Austria; 3 Department of Studies and Statistics for Integrated Research and Projects, Children’s Cancer Research Institute, Vienna, Austria; 4 Department of Paediatrics, St Anna Children’s Hospital, Medical University of Vienna, Vienna, Austria

**Keywords:** Paediatric cancer, Cancer immunotherapy

## Abstract

**Background:**

Short-term infusions of dinutuximab beta plus isotretinoin and cytokines administered in previous immunotherapy studies in neuroblastoma were associated with severe pain. Here, long-term, continuous infusion of single-agent dinutuximab beta was evaluated in patients with relapsed/refractory neuroblastoma.

**Methods:**

In this open-label, single-arm, Phase 2 study, patients with either refractory or relapsed high-risk neuroblastoma received dinutuximab beta by continuous infusion over 10 days of each cycle, for up to five cycles. The primary endpoint was objective response rate 24 weeks after the end of cycle 5. Secondary endpoints included adverse events, intravenous morphine use, best response, duration of response, and three-year progression-free and overall survival.

**Results:**

Of the 40 patients included, 38 had evaluable response. Objective response rate was 26% and best response rate 37%. Median duration of response was 238 days (IQR 108–290). Three-year progression-free and overall survival rates were 31% (95% CI 17–47) and 66% (95% CI 47–79), respectively. Prophylactic intravenous morphine use and duration of use decreased with increasing cycles. The most common grade 3 treatment-related adverse events were pain, diarrhea, and hypokalemia.

**Conclusion:**

Long-term continuous infusion of single-agent dinutuximab beta is tolerable and associated with clinically meaningful responses in patients with relapsed/refractory high-risk neuroblastoma.

**Clinical trial registration:**

The study is registered with ClinicalTrials.gov (NCT02743429) and EudraCT (2014-000588-42).

## Background

Neuroblastoma is the most common extracranial solid tumor of childhood and accounts for 12–15% of cancer-related deaths in children [1]. Approximately 50% of patients with neuroblastoma have high-risk disease [2], associated with three-year event-free survival (EFS) of only ~50%, despite intensive multimodal treatment [3, 4].

A randomized trial has demonstrated that therapy with an anti-GD_2_ antibody, interleukin-2 (IL-2), granulocyte-macrophage colony-stimulating factor (GM-CSF) and isotretinoin in the maintenance setting improves outcomes of patients with high-risk neuroblastoma (HR-NB) [5]. However, as GD_2_ is not only expressed on neuroblastoma cells, but also on sensory neuronal tissue [6], a common on-target side effect of anti-GD_2_ antibodies is neuropathic pain, which requires the co-administration of analgesic drugs, including intravenous morphine [7]. Another on-target side effect associated with anti-GD_2_ antibodies is neurotoxicity, which also requires careful management with supportive therapy [8].

A long-term, continuous infusion regimen for the anti-GD_2_ antibody dinutuximab beta was developed based on the clinical observation that decreasing the speed of the infusion reduced the incidence and severity of pain. In a single-center compassionate use program in patients with HR-NB, dinutuximab beta (100 mg/m^2^/cycle) infused continuously over 10 days combined with IL-2 and isotretinoin was associated with an acceptable safety profile, including low pain scores, a reduced need for intravenous morphine, and a low frequency of grade 3 and grade 4 adverse events [9].

In the HR-NBL1/SIOPEN study, where patients with HR-NB were given dinutuximab beta as a short, 8-h infusion with or without IL-2 in addition to isotretinoin, co-administration of IL-2 was found to increase toxicity, without further improving outcomes [10].

Differentiation therapy with isotretinoin has been the standard of care for patients with neuroblastoma for many years, based on the results of a randomized clinical study demonstrating its benefits on EFS [11]. However, long-term follow-up data indicated that isotretinoin had no significant effect on either EFS or overall survival (OS) over the longer term [12].

Here, we report the results of a multicenter clinical study investigating the activity and safety of single-agent dinutuximab beta (i.e., without IL-2 or isotretinoin) administered by long-term, continuous infusion in patients with relapsed/refractory HR-NB.

## Methods

### Study design and participants

This was a prospective, open-label, single-arm, multicenter, Phase 2 study (APN311-304) in which single-agent dinutuximab beta, administered by continuous, long-term infusion, was investigated in patients with relapsed/refractory HR-NB. Patients aged 1–21 years with neuroblastoma, according to the International Neuroblastoma Staging System (INSS) criteria [13], were eligible for inclusion if they had primary refractory Stage 4 disease or had relapsed after primary Stage 4 disease, or had developed distant metastases following primarily localized neuroblastoma, and their tumor burden was controlled using conventional therapy but with measurable disease still present. Patients who received previous anti-GD2 antibody therapies were eligible, if they were negative for anti-dinutuximab beta antibody prior to study entry.

### Procedures

Dinutuximab beta 10 mg/m^2^/day was administered as continuous infusion over the first 10 days of each 35-day cycle (Supplementary Fig. S1) for up to five cycles (in the absence of disease progression). Dinutuximab beta was planned to be administered in the hospital setting in each cycle, but if well tolerated, it could be given in an outpatient setting from day 5 of cycle 1.

Pain management with oral gabapentin, and morphine was administered according to the World Health Organization guidelines. Based on the individual patient’s pain tolerance, subsequent treatment days within a cycle as well as subsequent treatment cycles could be started with an adapted intravenous morphine dose. Prophylaxis for fever was metamizole, acetaminophen, or ibuprofen, according to institutional standards. All patients received hydration during administration of dinutuximab beta to prevent hypotension.

Chemotherapy, radiotherapy, hormonal anticancer therapy, or experimental anticancer medications were not permitted during the study or the 12- and 24-week follow-up periods. The use of intravenous immunoglobulin was discouraged during dinutuximab beta treatment.

### Outcomes

The primary endpoint of the study was the objective response rate (ORR) 24 weeks after the end of cycle 5, defined as the proportion of patients with a complete or partial response based on the International Neuroblastoma Response Criteria (INRC) [13]. The secondary endpoints were duration of response, three-year progression-free survival (PFS) and OS rates, response at each tumor evaluation, best response, safety and tolerability, pharmacokinetics, pharmacodynamics, and immunogenicity.

Tumor assessments according to the INRC, including magnetic resonance imaging (MRI), computed tomography (CT) and ^123^I-metaiodobenzylguanidine (mIBG) scan, were undertaken in the last 2 weeks of cycle 2, at the end of cycle 5 (or at the end of treatment if discontinued before cycle 5), 12 and 24 weeks after completing study treatment, and then every three months for 3 years. All assessments were centrally reviewed by a blinded review committee.

Safety and tolerability assessments included changes in vital signs and clinical laboratory parameters, pain intensity assessment scores (0 [no pain] to 10 [unbearable pain] on a validated pain scale), and the need for, amount, and duration of intravenous morphine. In addition, adverse events were recorded and graded using the Common Terminology Criteria for Adverse Events, version 4.0.

Pharmacokinetic assessments included determining dinutuximab beta serum concentrations in cycle 1 at baseline, the end of infusion, and on days 15, 22, 29, and 35 [14]. Pharmacodynamic assessments included antibody-dependent cell-mediated cytotoxicity, and complement-dependent cytotoxicity [15]. The sampling time points for pharmacokinetic and pharmacodynamic parameters are shown in Supplementary Fig. S1. Immunogenicity was assessed by the presence of human anti-chimeric antibodies (HACA) [16, 17].

### Statistical analysis

For the primary endpoint, a sample size of 40 evaluable patients would provide at least 80% power to reject the null hypothesis of an ORR of <10% if the true ORR observed was 25%, which is ~20% higher than the estimated historical response rate [18, 19] in this patient population (overall one-sided significance level 0.05, exact binomial test).

The primary endpoint was analyzed using the exact binomial test (one-sided) using SAS Version 9.4, with an overall significance level of 0.05, corresponding to an alpha of 0.1 for a two-sided test. Post-hoc analyses of the primary endpoint were also undertaken: (1) in patients with bone marrow involvement, (2) in patients with relapsed versus refractory disease, and (3) based on an updated INRC definition of ORR that includes minor response [20]. Fisher’s exact test (two-sided) was used to determine if there was a significant association between disease status at study entry and response.

Survival probabilities were estimated using Kaplan–Meier methods, and statistical comparisons were performed using the log-rank test (assuming proportional hazard). Three-year survival rates are shown as means and 95% confidence interval (CI). A post-hoc analysis was conducted to evaluate PFS and OS in patients stratified by disease status at study entry (relapsed versus refractory disease).

Pharmacokinetic, pharmacodynamic and immunogenicity data are presented as mean and standard deviation (SD). Differences between groups were calculated using a Mann–Whitney rank sum test [21].

## Results

Between March 30, 2015, and December 7, 2018, 40 patients with relapsed or refractory HR-NB were recruited, all of whom received dinutuximab beta and were included in the safety analysis set. Baseline characteristics are shown in Table 1. Tumor responses were evaluated in 38 patients (full analysis set): 21 with relapsed and 17 with refractory disease. Three patients received previous anti-GD2 antibody therapy.

**Table 1:** Baseline patient characteristics.

	Patients (*N* = 40)
Age	
Median (IQR) age at initial diagnosis, years [mean ± SD]	3.5 (2.4–5.4) [3.9 ± 2.3]
Median (IQR) age at informed consent, years [mean ± SD]	6.0 (4–7.5) [6.4 ± 3.5]
Sex	
Male	28 (70)
Female	12 (30)
Race, white	40 (100)
Disease status	
Relapsed	23 (57)
Refractory	17 (43)
Relapses since initial diagnosis	
None	17 (43)
1	19 (48)
2	4 (10)
INSS stage at initial diagnosis	
1	2 (5)
2	0
3	2 (5)
4	36 (90)
*MYCN* amplification	
No	32 (80)
Yes	7 (18)
Missing	1 (3)
Bone marrow involvement	14 (35)
Soft tissue involvement	17 (43)

*INSS* International Neuroblastoma Staging System, *IQR* interquartile range, *SD* standard deviation.

Data are *n* (%) unless stated otherwise.

**Fig. 1:**
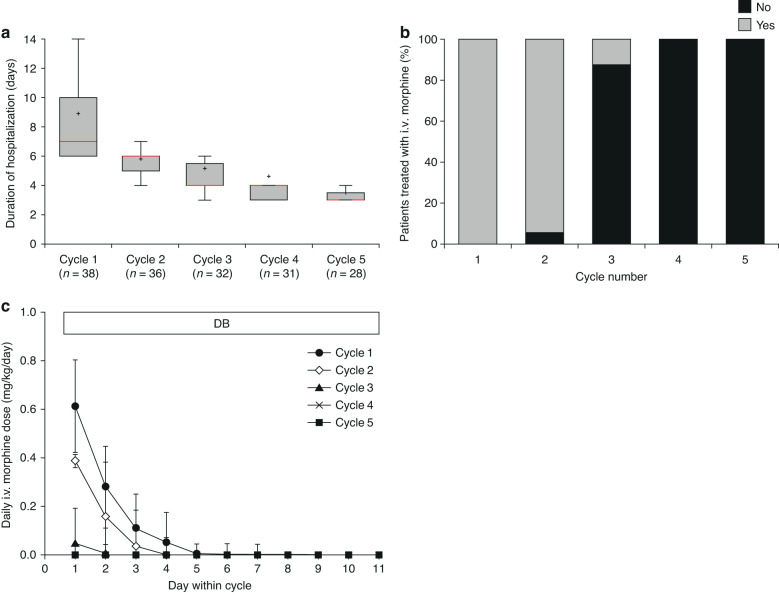
Duration of hospitalization and use of intravenous morphine over time. The duration of hospitalization for antibody treatment (**a**), proportion of patients requiring intravenous morphine during five treatment cycles with single-agent dinutuximab beta (**b**) and the daily dose of intravenous morphine required (**c**). **a** The box limits are the 1st and 3rd quartile with the median value indicated by the red line and the whiskers represent the most extreme points (1.5 × IQR); outliers are not displayed. + indicates the mean value. The duration of hospitalization was calculated as the date of discharge-date of admission +1. If readmission occurs within 11 days of day 1 of the cycle, the date of readmission is added. **b** The data represent the mean and in panel c the mean ± SD. DB dinutuximab beta, IQR interquartile range, i.v. intravenous, SD standard deviation.

Cycle 1 of dinutuximab beta was completed by 39 patients (mean cumulative dose per cycle: 91.5 mg/m^2^ [SD 15.7]). The number of patients receiving subsequent cycles of dinutuximab beta decreased due to progressive disease. Patients were hospitalized for the first 7 days (median, interquartile range [IQR] 6–10) of cycle 1, reducing to 3 days (median, IQR 3–3.25) in cycle 5 (Fig. 1a), indicating that an increasing proportion of the 10-day continuous infusion could be administered in an outpatient setting.

All patients received intravenous morphine on day 1 of cycle 1 at a mean dose of 0.611 mg/kg (SD 0.191) (Fig. 1b). The dose of morphine required decreased within the cycles, and with subsequent cycles (Fig. 1c). The mean duration of morphine treatment also generally decreased with subsequent cycles, from 3.2 days (SD 1.4) in cycle 1, to 2.2 days (SD 0.9), and 0.3 days (SD 0.8) in cycles 2 and 3, respectively, and 0 days in cycles 4–5.

During cycle 1, 92% of patients reported pain at rest, compared with ~22% during cycle 5 (Supplementary Table S1). In patients who experienced pain, mean pain scores were low and decreased between cycles 1 and 2: from 1.01 to 0.30 at rest, and from 1.45 to 0.28 at stress.

Overall, 13 (32%) patients who received dinutuximab beta had treatment-related grade 3 toxicities (Table 2); no grade 4 or 5 events were reported. The most common grade 3 toxicities were neuropathic pain, diarrhea, and hypokalemia. No cases of grade 3 hypotension, capillary leak syndrome, hypersensitivity, bronchospasm, allergic reaction, or neurotoxicity were reported.

**Table 2:** Grade 3 treatment-related, treatment-emergent adverse events.

	Grade 3
Neuropathic pain	7 (18)
Diarrhea	2 (5)
Hypokalemia	2 (5)
Hyperbilirubinemia	1 (2)
Hypoglycemia	1 (2)
Hyponatremia	1 (2)
Pruritis	1 (2)
Pyrexia	1 (2)
Tachycardia	1 (2)

Data are *n* (%). No treatment-related grade 4 adverse events were reported, and there were no deaths due to treatment-related adverse events.

The ORR 24 weeks after the end of cycle 5 of dinutuximab beta was 26% (10/38), which was higher than the null hypothesis of 10% (*P* = 0.0034). When minor responses were also considered, the ORR was 32% (12/38). Nine responding patients were refractory and three had relapsed disease, indicating a statistically significant association between response rate and disease status at study entry (*P* = 0.0159). The ORR based on the best overall response up to 24 weeks after the end of cycle 5 was 37% (14/38, *P* < 0.0001), and 53% (20/38) when minor responses were also considered (Table 3 and Supplementary Table S2). In the post-hoc analysis of the 14 patients with bone marrow involvement at baseline, 12 patients achieved a complete response and one a partial response, indicating a response rate of 93%. In the full analysis set, the median duration of response was 238.0 days (IQR 108–290). The best response rate according to the INRC components varied between assessments and was highest in the bone marrow compartment (93%) followed by the osteomedullary compartment assessed using mIBG scans (53%) and the soft tissue compartment assessed by CT/MRT scans (17%) (Supplementary Tables S3–5).

**Table 3:** Treatment response in 38 evaluable patients with relapsed or refractory neuroblastoma.

	Evaluation timepoint				
	During treatment		Time after treatment		
	Cycle 2	Cycle 5 (EoT)	12 weeks	24 weeks (EoS)	Best response
All patients (*n* = 38)					
Complete responses	0	2	3	4	4
Partial responses	10	9	6	6	10
Objective response rate*	10 (26%)	11 (29%)	9 (24%)	10 (26%)	14 (37%)
Minor responses	8	6	3	2	6
Updated objective response rate^§^	18 (47%)	17 (45%)	12 (32%)	12 (32%)	20 (53%)
Relapsed (*n* = 21)					
Complete responses	0	1	2	2	2
Partial responses	5	4	1	1	4
Objective response rate*	5 (24%)	5 (24%)	3 (14%)	3 (14%)	6 (29%)
Minor responses	3	2	0	0	3
Updated objective response rate^§^	8 (38%)	7 (33%)	3 (14%)	3 (14%)	9 (43%)
Refractory (*n* = 17)					
Complete responses	0	1	1	2	2
Partial responses	5	5	5	5	6
Objective response rate*	5 (29%)	6 (35%)	6 (35%)	7 (41%)	8 (47%)
Minor responses	5	4	3	2	3
Updated objective response rate^§^	10 (59%)	10 (59%)	9 (53%)	9 (53%)	11 (65%)

*EoS* end of study, *EoT* end of treatment, *INRC* International Neuroblastoma Response Criteria.

*Objective response rate includes patients with complete and partial response. ^§^The updated objective response rate includes patients with complete, partial and minor response following an update of the INRC during the study (in 2017).

**Fig. 2:**
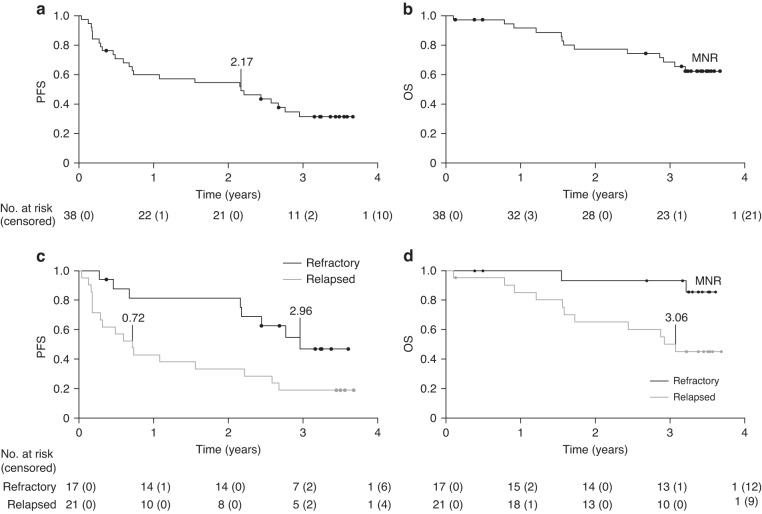
Median three-year survival with single-agent dinutuximab beta. Kaplan–Meier estimates for progression-free survival (**a**), overall survival (**b**), progression-free survival by disease status (**c**), and overall survival by disease status (**d**). OS overall survival, PFS progression-free survival, MNR median not reached, y years.

The three-year PFS rate was 31% (95% CI 0.17–0.47) and OS rate was 66% (95% CI 0.47–0.79) in the full analysis set (*n* = 38) (Fig. 2). When patients were analysed according to their disease status at study entry, three-year survival rates were lower for patients with relapsed disease compared with those with refractory disease (three-year PFS rate: 19% [95% CI 2–36] vs 47% [95% CI 21–73, *P* = 0.015]; three-year OS rate: 50% [95% CI 28–72] vs 93% [95% CI 81–1, *P* = 0.015]) (Fig. 2).

**Fig. 3:**
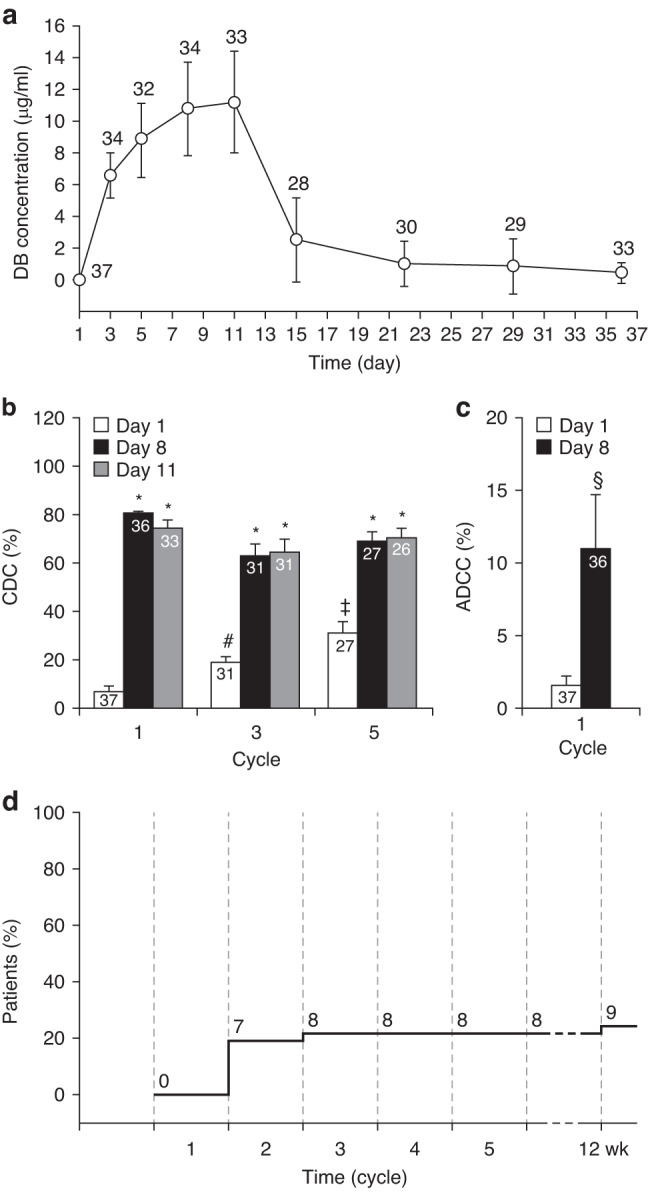
Pharmacokinetics and pharmacodynamics of dinutuximab beta. Plasma concentration-time curve for dinutuximab beta administered by long-term, continuous infusion over 10 days (**a**). Pharmacodynamic effects of dinutuximab beta on complement-dependent cytotoxicity in cycles 1, 3 and 5 (**b**) and antibody-dependent cellular cytotoxicity in cycle 1 (**c**). Cumulative incidence of human anti-chimeric antibody response after dinutuximab beta treatment (**d**). **a**–**c** Data represent mean ± SD. All *P* values are versus values on day 1 of cycle 1 using the Mann–Whitney rank sum test: *P < 0.0001; ^#^*P* = 0.0057; ^‡^*P* < 0.0001; ^§^*P* = 0.0208.

In total, 35 patients were evaluable for the pharmacokinetic analysis—one patient was excluded due to missing samples, and two developed HACAs during cycle 1. The concentration-time curve for dinutuximab beta administered as long-term infusion (Fig. 3a) showed a mean peak serum concentration at the end of infusion of 11.2 µg/ml (SD 3.2) during cycle 1, followed by rapid clearance with an alpha half-life of 2.3 days (SD 2.1) and a terminal beta half-life of 7.8 days (SD 2.9).

In the pharmacodynamic analysis (*n* = 35), serum levels of dinutuximab beta translated into significantly increased complement-dependent (11-fold; Fig. 3b) and antibody-dependent cellular cytotoxicity (seven-fold; Fig. 3c) on day 8 in cycle 1 compared with pre-treatment (day 1) in cycle 1 (Fig. 3b). The complement activity was significantly increased at trough dinutuximab beta levels preceding subsequent cycles (day 1 of cycles 3 and 5 compared with pre-treatment [day 1] in cycle 1; Fig. 3b), indicating immunological activity in patients treated with dinutuximab beta over the duration of the treatment period. HACA response at the end of treatment was found in eight of 37 (22%) patients, and the cumulative incidence over time shows that the response occurred during the first two cycles. There was no correlation between the dinutuximab beta peak or trough concentration, or the area under the curve in cycle 1, and HACA response or survival (data not shown).

## Discussion

This open-label, Phase 2 study evaluated long-term continuous infusion of dinutuximab beta without IL-2 or isotretinoin in patients with relapsed/refractory HR-NB. The best overall response rate (37%), three-year PFS rate (31%), three-year OS rate (66%), and median duration of response (238 days) indicate the clinical activity and efficacy of single-agent dinutuximab beta. Interestingly, the response and survival rates in patients with refractory disease were signifcantly higher than in patients with relapsed disease, suggesting that future trials should be stratified by separating these subgroups.

Response and survival are similar to those achieved when dinutuximab beta was administered in combination with IL-2 and isotretinoin in a similar patient population (ORR 41%, three-year PFS rate 33%, three-year OS rate 48%) [9], suggesting no additional benefit for IL-2 and isotretinoin co-administration. However, ideally, a randomized trial should be conducted comparing dinutuximab beta with or without IL-2 and isotretinoin in this population, similar to the front-line setting [10]. Similar tumor responses were also reported with the humanized anti-GD_2_ antibody naxitamab co-administered with GM-CSF in a Phase 1/2 study in patients with primary or secondary refractory neuroblastoma [22]. In an interim analysis including 38 patients, the ORR was 34% and the duration of response was at least 6 months [22], which is similar to the findings of the current study in the absence of concomitant cytokines. In another Phase 2 study in patients with HR-NB with bone and/or bone marrow disease, naxitamab plus GM-CSF resulted in an ORR of 68% [23]. In a subgroup of 14 patients with bone marrow involvement in the current study, the response rate was 93%, suggesting that anti-GD_2_ immunotherapy has particularly high activity against bone marrow disease.

Based on this study, single-agent dinutuximab beta administered by long-term infusion is associated with a better safety profile than that reported in a study evaluating dinutuximab beta combined with IL-2 [9]. This is true for both the on-target side effects of pain and neurotoxicity, as well as inflammatory side effects. The use of intravenous morphine was not required by any patient during treatment cycles 4 and 5 of single-agent dinutuximab beta; in contrast, it was necessary in all five cycles when dinutuximab beta was combined with IL-2 [9]. Given the observation that >90% of patients did not require intravenous morphine after cycle 2 in our study, it is likely that >90% of patients could receive treatment in the outpatient setting from cycle 3 onwards. In a similar study evaluating the use of dinutuximab beta combined with isotretinoin, the proportion of patients who did not require intravenous morphine after cycle 2 was found to be 100% [24]. The incidence of grade 3 pain observed in this study (18%) was lower than for dinutuximab beta plus IL-2 in previous studies (26–38%) [9, 10]. Furthermore, there were no cases of grade 3 and 4 capillary leak syndrome, hypersensitivity reactions, bronchospasm, or allergic reactions with single-agent dinutuximab beta, while in studies where it was co-administered with IL-2, the incidences were 13–15%, 2–21%, 8%, and 15%, respectively [9, 10].

The pharmacokinetics of dinutuximab beta were similar when administered alone here or co-administered with IL-2 in an earlier study, with mean peak plasma concentration of 11.2 µg/ml (SD 3.2) and 12.56 µg/ml (SD 0.68), respectively [17]. Similarly, the incidence of HACA responses and the levels of complement-dependent and antibody-dependent cellular cytotoxicity responses were also comparable with or without IL-2 [25]. These results demonstrate that there is no loss of biological activity in the absence of IL-2. The absence of a correlation between the peak/trough concentration or the area under the curve of dinutuximab beta in cycle 1, and HACA response or survival is in contrast to previous reports for dinutuximab [26, 27], underlining the molecular differences between these antibodies.

Limitations of the study include the heterogeneity of the enrolled patient population regarding their second-line therapy. The study was conducted to determine the activity of single-agent dinutuximab beta in patients with relapsed/refractory disease who had responded to a second-line therapy that was not predefined in the study protocol. Therefore, as all patients had to be in stable disease to be eligible, the responses must be interpreted with caution. In contrast, many other Phase 2 studies in neuroblastoma included patients with progressive disease at study entry.

Despite these limitations, our findings demonstrate that single-agent dinutuximab beta (i.e., without IL-2 and isotretinoin) administered by long-term, continuous infusion in patients with relapsed/refractory HR-NB was associated with improved tolerability and clinically significant and durable response rates that translated into encouraging three-year PFS and OS rates.

### Supplementary information

Figure S1: Treatment and assessment schedule

Table S1: Pain intensity

Table S2: Individual treatment responses in 38 evaluable patients with relapsed or refractory neuroblastoma

Table S3: Response according to INRC components: mIBG response

Table S4: Response according to INRC components: CT/MRI response (according to RECIST)

Table S5: Response according to INRC components: bone marrow response

## Data Availability

The data generated in this study are available upon request from the corresponding author.
