# Supplementary figures and images for: Long-term, continuous infusion of single-agent dinutuximab beta for relapsed/refractory neuroblastoma: an open-label, single-arm, Phase 2 study
Source: Br J Cancer. 2023 Oct 10;129(11):1780–6. doi: 10.1038/s41416-023-02457-x (PMC10667538; doi:10.1038/s41416-023-02457-x)

**Figure S1: Treatment and assessment schedule**

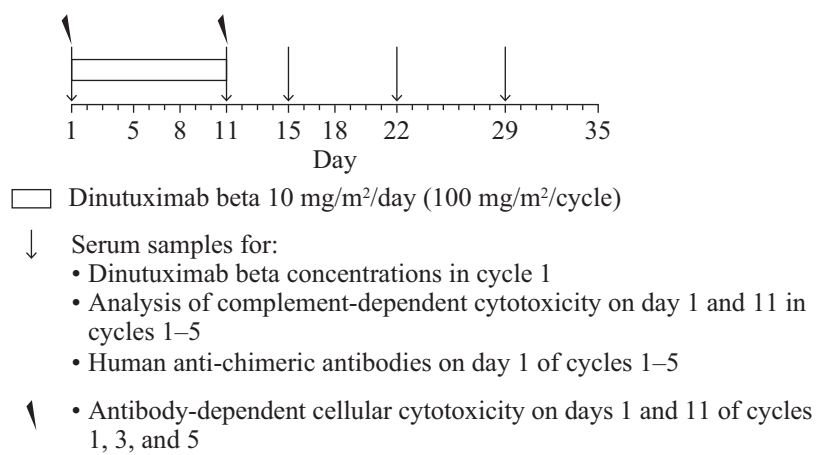

Supplement: Supplementary file 1 — Figure S1: Treatment and assessment schedule [file 41416_2023_2457_MOESM1_ESM.pdf]
