# Supplementary material for: Long-term, continuous infusion of single-agent dinutuximab beta for relapsed/refractory neuroblastoma: an open-label, single-arm, Phase 2 study
Source: Br J Cancer. 2023 Oct 10;129(11):1780–6. doi: 10.1038/s41416-023-02457-x (PMC10667538; doi:10.1038/s41416-023-02457-x)
Supplement: Supplementary file 2 — Table S1: Pain intensity [file 41416_2023_2457_MOESM2_ESM.docx]

**Table S1: Pain intensity**

|  | **Cycle 1** | **Cycle 2** | **Cycle 3** | **Cycle 4** | **Cycle 5** |
| --- | --- | --- | --- | --- | --- |
| **At rest** | | | | | |
| n | 38 | 35 | 31 | 30 | 27 |
| Patients with pain, n (%*) | 35 (92) | 7 (20) | 6 (19) | 6 (20) | 6 (22) |
| Mean^#^ ± SD | 1.01 ± 0.63 | 0.30 ± 0.18 | 0.59 ± 0.43 | 0.78 ± 0.52 | 0.62 ± 0.44 |
| Median (IQR) | 1.00 (0.40–1.38) | 0.27 (0.20–0.38) | 0.53 (0.22–1.05) | 0.74 (0.33–1.00) | 0.46 (0.33–0.89) |
| **At stress** | | | | | |
| n | 31 | 28 | 25 | 23 | 22 |
| Patients with pain, n (%*) | 25 (81) | 5 (18) | 4 (16) | 3 (13) | 5 (23) |
| Mean^#^ ± SD | 1.45 ± 1.3 | 0.28 ± 0.15 | 0.82 ± 0.59 | 0.88 ± 0.22 | 1.02 ± 1.88 |
| Median (IQR) | 1.21 (0.75–1.80) | 0.33 (0.18–0.40) | 0.78 (0.32–1.32) | 1.00 (0.63–1.00) | 0.38 (0.30–1.20) |

Pain was assessed using age-adapted scales with scores ranging from 0 (no pain) to 10 (unbearable pain), where a score of 5 warrants therapeutic intervention. *Proportion of patients with pain in the respective cycle (patients with pain/patients with pain assessment). ^#^For each patient, the mean score of all pain assessments within a given cycle was used. IQR,interquartile range; SD,standard deviation.
