# Supplementary material for: Long-term, continuous infusion of single-agent dinutuximab beta for relapsed/refractory neuroblastoma: an open-label, single-arm, Phase 2 study
Source: Br J Cancer. 2023 Oct 10;129(11):1780–6. doi: 10.1038/s41416-023-02457-x (PMC10667538; doi:10.1038/s41416-023-02457-x)
Supplement: Supplementary file 3 — Table S2: Individual treatment responses in 38 evaluable patients with relapsed or refractory neuroblastoma [file 41416_2023_2457_MOESM3_ESM.docx]

Table S2: Individual treatment responses in 38 evaluable patients with relapsed or refractory neuroblastoma

|  |  | **Evaluation timepoint** | | | |  |
| --- | --- | --- | --- | --- | --- | --- |
|  |  | **During treatment** | | **Time after treatment** | |  |
| **Patient** | **Status at study entry** | **Cycle 2** | **Cycle 5 (EoT)** | **12 weeks** | **24 weeks (EoS)** | **Best response** |
| 01 | Refractory | PR | PR | PR | CR | CR |
| 02* | Relapsed | MR | MR | NT |  | MR |
| 03 | Relapsed | SD | SD | SD | SD | SD |
| 04 | Relapsed | PR | PR | PR | NE^#^ | PR |
| 05 | Refractory | MR | MR | MR | PR | PR |
| 06 | Refractory | PR | CR | CR | CR | CR |
| 07* | Relapsed | PD |  |  |  | PD |
| 08 | Refractory | PD |  |  |  | PD |
| 09 | Relapsed | PD |  |  |  | PD |
| 10 | Refractory | SD | SD | SD | SD | SD |
| 11 | Relapsed | PR | SD | NT |  | PR |
| 12 | Refractory | MR | MR | MR | MR | MR |
| 13 | Relapsed | PD |  |  |  | PD |
| 14 | Refractory | SD | PD |  |  | SD |
| 15^‡^ | Refractory | SD | SD | SD | SD | SD |
| 16 | Relapsed | SD | PD | PD |  | SD |
| 17 | Relapsed | PR | CR | CR | CR | CR |
| 18 | Refractory | PR | PR | ND | PR | PR |
| 19 | Relapsed | SD | SD | SD | NT | SD |
| 20* | Relapsed | PR | PR | ND. | PR | PR |
| 21 | Relapsed | PD |  |  |  | PD |
| 22 | Refractory | SD | SD^§^ |  |  | SD |
| 23 | Relapsed | PD |  |  |  | PD |
| 24* | Refractory | PR | PR | PR | PR | PR |
| 25 | Relapsed | MR | MR | NT |  | MR |
| 26 | Relapsed | MR | PD |  |  | MR |
| 27* | Relapsed | PR | PR | PD | PD | PR |
| 28^c^ | Relapsed | SD | SD | PD |  | SD |
| 29* | Relapsed | SD | PR | CR | CR | CR |
| 30 | Refractory | PR | PR | PR | PR | PR |
| 31*^,¶^ | Relapsed | SD | PD |  |  | SD |
| 32* | Refractory | SD | SD | PR | PR | PR |
| 33 | Refractory | MR | PR | PR | NT | PR |
| 34 | Relapsed | SD | SD | SD | SD | SD |
| 35* | Refractory | MR | MR | MR | MR | MR |
| 36 | Refractory | MR | MR | PD |  | MR |
| 37 | Relapsed | PD |  |  |  | PD |
| 38^‡^ | Refractory | SD | SD | SD | SD | SD |

*HACA positive. ^#^Assessment was done, but it was not evaluable. ^‡^Received previous anti-GD2 antibody therapy. ^§^Premature discontinuation after 4 cycles. ^¶^Developed HACA response 12 weeks after completion of study treatment (late responder). CR, complete response; EoS, end of study; EoT, end of treatment; HACA, human anti-chimeric antibodies; MR, minor response; ND, not determined; NE, not evaluable; NT, new treatment started before EoS; PD, progressive disease; PR, partial response; SD, stable disease.
