# Supplementary material for: Long-term, continuous infusion of single-agent dinutuximab beta for relapsed/refractory neuroblastoma: an open-label, single-arm, Phase 2 study
Source: Br J Cancer. 2023 Oct 10;129(11):1780–6. doi: 10.1038/s41416-023-02457-x (PMC10667538; doi:10.1038/s41416-023-02457-x)
Supplement: Supplementary file 4 — Table S3: Response according to INRC components: mIBG response [file 41416_2023_2457_MOESM4_ESM.docx]

**Table S3: Response according to INRC components: mIBG response***

| **Patient** | **Baseline** | **Mid evaluation** |  | **End of treatment** |  | **12-week FU** |  | **24-week FU** |  | **Best response** |
| --- | --- | --- | --- | --- | --- | --- | --- | --- | --- | --- |
|  | **SIOPEN score** | **SIOPEN score** |  | **SIOPEN score** |  | **SIOPEN score** |  | **SIOPEN score** |  |  |
| 1 | 13 |  | PR | 9 | PR | 1 | PR | 0 | CR | CR |
| 2 | 1 + 1 soft tissue | 0 + 1 soft tissue | PR | 0 + 1 soft tissue | PR |  |  | 0 | CR | CR |
| 3 | 2 | 2 | SD | 2 | SD | 2 | SD | 2 | SD | SD |
| 4 | 3 |  | PR | 2 | PR | 2 | PR | 2 | PR | PR |
| 5 | 2 + 1 soft tissue | 2 + 1 soft tissue | SD | 2 + 1 soft tissue | SD | 2 + 0 soft tissue | PR | 2 + 0 soft tissue | PR | PR |
| 6 | 13 | 1 | PR | 0 | CR | 0 | CR | 0 | CR | CR |
| 7 | 0 + 2 soft tissue | 1 + 2 soft tissue | PD |  |  |  |  |  |  | PD |
| 8 | 16 | 19 | PD |  |  |  |  |  |  | PD |
| 9 | 5 + 3 soft tissue | 7 + 3 soft tissue | PD |  |  |  |  |  |  | PD |
| 10 | 0 + 1 soft tissue | 0 + 1 soft tissue | SD | 0+1 soft tissue | SD | 0 + 1 soft tissue | SD | 0 + 1 soft tissue | SD | SD |
| 11 | 34 + 1 soft tissue | 1 | PR | 34 + 1 soft tissue | SD |  |  |  |  | PR |
| 12 | 14 + 1 soft tissue | 9 + 1 soft tissue | PR | 9 + 1 soft tissue | PR | 9 + 1 soft tissue | PR | 9 + 1 soft tissue | PR | PR |
| 13 | 0 + 2 soft tissue | 0 + 3 soft tissue | PD |  |  |  |  |  |  | PD |
| 14 | 33 + 5 soft tissue | 33 + 5 soft tissue | SD | 33 + 5 soft tissue | SD |  |  |  |  | SD |
| 15 | 1 | 1 | SD | 1 | SD | 1 | SD | 1 | SD | SD |
| 16 | 0 | 0 | NE | 0 + 1 soft tissue | PD | 1 + 1 soft tissue | PD |  |  | PD |
| 17 | 1 +1 soft tissue | 0 + 1 soft tissue | PR | 0 | CR | 0 | CR | 0 | CR | CR |
| 18 | 28 | 12 | PR | 12 | PR |  |  |  |  | PR |
| 19 | 2 | 2 | SD | 2 | SD | 2 | SD |  |  | SD |
| 20 | 13 | 2 | PR | 3 | PR |  |  |  |  | PR |
| 21 | 0 | ND | ND |  |  |  |  |  |  | NE |
| 22 | 0 + 1 soft tissue | 0 + 1 soft tissue | SD | 0 + 1 soft tissue | SD |  |  |  |  | SD |
| 23 | 0 | 2 | PD |  |  |  |  |  |  | PD |
| 24 | 10 | 4 | PR | 4 | PR | 3 | PR | 2 | PR | CR |
| 25 | 36 | 36 | SD | 36 | SD |  |  | 1 | PR | PR |
| 26 | 1 + 2 soft tissue | 1 + 2 soft tissue | SD |  | ND |  |  |  |  | SD |
| 27 | 8 | 1 | PR | 1 | PR | 3 | PR | 2 | PR | PR |
| 28 | 0 + 1 soft tissue | 0 + 1 soft tissue | SD | 0 + 1 soft tissue | SD | 3 + 1 soft tissue | PD |  |  | SD |
| 29 | 2 | 2 | SD | 1 | PR | 0 | CR | 0 | CR | CR |
| 30 | 53 | 9 | PR | 7 | PR | 8 | PR | 6 | PR | PR |
| 31 | 1 | 1 | SD | 4 | PD |  |  |  |  | SD |
| 32 | 14 | 6 | PR | 9 | PR | 6 | PR | 8 | PR | PR |
| 33 | 3 + 2 soft tissue | 0 + 2 soft tissue | PR | 0 + 1 soft tissue | PR | 0 + 1 soft tissue | PR | 0 + 1 soft tissue | PR | PR |
| 34 | 3 + soft tissue | 3 + soft tissue | SD | 2 + soft tissue | PR | 2 + soft tissue | PR | 2 + soft tissue | PR | PR |
| 35 | 0 + 1 soft tissue | 0 | CR | 0 | CR | 0 | CR | 0 | CR | CR |
| 36 | 0 + 1 soft tissue | 0 + 1 soft tissue | SD | 0 + 1 soft tissue | SD | 0 + 1 soft tissue | SD | 0 + 1 soft tissue | SD | SD |
| 37 | 0 | 0 | NE |  |  |  |  |  |  | NE |
| 38 | 1 | 1 | SD | 1 | SD | 1 | SD | 1 | SD | SD |
|  |  |  |  |  |  |  |  |  |  |  |
|  |  |  |  |  |  |  |  |  | CR: | 7 |
|  |  |  |  |  |  |  |  |  | PR: | 12 |
|  |  |  |  |  |  |  |  |  | SD: | 11 |
|  |  |  |  |  |  |  |  |  | Response rate | 19/36 = 53% |
|  |  |  |  |  |  |  |  |  | CR rate | 7/36 = 19% |

*mIBG scans were assessed using the SIOPEN score: ^123^I-mIBG scintigraphy in neuroblastoma: development of a SIOPEN semi-quantitative reporting, method by an international panel V. Lewington, B. Lambert, U. Poetschger, Z. Bar Sever, E. Giammarile, A. J. B. McEwan, Rita Castellani, T. Lynch, B. Shulkin, M. Drobics, A. Staudenherz, R. Ladenstein Eur J Nucl Med Mol Imaging (2017) 44:234–241, DOI 10.1007/s00259-016-3516-0. Blue indicates CR and green PR. CR, complete response; FU, follow-up; INRC, International Neuroblastoma Response Criteria; mIBG, metaiodobenzylguanidine; ND, not determined; NE, not evaluable; PR, partial response; SD, stable disease; SIOPEN, International Society of Paediatric Oncology Europe Neuroblastoma Group.
