# Supplementary material for: Long-term, continuous infusion of single-agent dinutuximab beta for relapsed/refractory neuroblastoma: an open-label, single-arm, Phase 2 study
Source: Br J Cancer. 2023 Oct 10;129(11):1780–6. doi: 10.1038/s41416-023-02457-x (PMC10667538; doi:10.1038/s41416-023-02457-x)
Supplement: Supplementary file 5 — Table S4: Response according to INRC components: CT/MRI response (according to RECIST) [file 41416_2023_2457_MOESM5_ESM.docx]

**Table S4: Response according to INRC components: CT/MRI response (according to RECIST)**

| **Patient** | **Baseline** | **Mid evaluation** | **End of treatment** | **12-week FU** | **24-week FU** | **Best response** |
| --- | --- | --- | --- | --- | --- | --- |
| 1 | NED | NED | NED | NED | NED | NE |
| 2 | celiac trunc  1.4 x 1.4 cm | celiac trunc  1.2 x 1.2 cm | celiac trunc  1.2 x 1.2 cm | ND | CR | SD |
| 3 | NED | NED | NED | NED | NED | NE |
| 4 | NED | NED | NED | NED | NED | NE |
| 5 | ri kidney 11 x 16 mm;  paraaortic 12 x 7 mm | ri kidney 11 x 16 mm;  paraaortic 12 x 7 mm | ri kidney 11 x 16 mm;  paraaortic 12 x 7 mm | ri kidney 5 x 11 mm;  paraortic 6 x 6 mm | ri kidney 5 x 5 mm;  paraaortic 10 x 4 mm | PR |
| 6 | NED | NED | NED | NED | NED | NE |
| 7 | kidneys 14 x 13 mm;  le kidney 16 x 13 mm | kidneys 14 x 13 mm;  le kidney 16 x 13 mm;  new lesion: re kidney  8 x 8 mm |  |  |  | PD |
| 8 | pulmonary 5 x 5 mm;  2^nd^ sacral vertebra 2 2.5 x 1.1 x 1.0 cm | pulmonary 5 x 5 mm;  2^nd^ sacral vertebra 2.5 x 1.1 x 1.0 cm;  new lesion 5^th^ lumbar vertebra |  |  |  | PD |
| 9 | le kidney 1.2 x 1.0 cm;  5^th^ lumbar vertebra 1.8 x 1.2 x 2.2 cm;  pelvis 3.3 x 6.6 x 7.6 cm | le kidney 1.3 x 1.4 cm;  5^th^ lumbar vertebra 5 1.8 x 1.2 x 2.2 cm;  pelvis 4.5 x 6.5 x 7.6 cm |  |  |  | PD |
| 10 | le kidney 1.5 x 0.8 cm;  le adrenal 1.1 x 1.0 cm | le kidney 1.5 x 0.8 cm;  le adrenal 1.1 x 1.0 cm | le kidney 1.5 x 0.8 cm;  le adrenal 1.1 x 1.0 cm | le kidney 1.5 x 0.8 cm;  le adrenal 1.1 x 1.0 cm | le kidney 1.5 x 0.8 cm;  le adrenal 1.1 x 1.0 cm | SD |
| 11 | NED | NED | NED |  |  | NE |
| 12 | paraortal 1.1 x 1.9 cm;  lymphnode pelvis 1.6 cm, os frontale; | paraortal 1.1 x 1.9 cm,  lymphnode pelvis 1.6 cm; os frontale; | paraortal 1,1 x 1,9 cm;  lymphnode pelvis 1.6 cm; os frontale | paraortal 1,1 x 1,9 cm;  lymphnode pelvis 1.6 cm; os frontale | paraortal 1.1 x 1.9 cm;  lymphnode pelvis 1.6 cm; os frontale | SD |
| 13 | liver lesions seg 6 + 7 | liver lesions seg 6 + 7, CNS lesion new |  |  |  | PD |
| 14 | supra/infraclavicular le 1.3 cm;  thoracic lesion; paraaortic lesion | supra/infraclavicular le 1.3 cm;  thoracic lesion, paraaortic lesion | supra/infraclavicular le 2.5 cm; thoracic lesion, paraaortic lesion |  |  | PD |
| 15 | NED | NED | NED | NED | NED | NE |
| 16 | intracerebral  1.4 x 0.6 cm | intracerebral  1.4 x 0.6 cm | intracerebral  1.4 x 0.6 cm,  4^th^ lumbar vertebra 2.4 x 1.4 cm |  |  | PD |
| 17 | retroperitoneal  4.1 x 1.9 cm | retroperitoneal  not measurable | no lesion | no lesion | no lesion | CR |
| 18 | NED | NED | NED |  |  | SD |
| 19 | NED | NED | NED | NED | NED | NE |
| 20 | NED | NED | NED |  |  | NE |
| 21 | le kidney 1.4 x 1.3 cm | le kidney 10 x 7,5 cm |  |  |  | PD |
| 22 | liver lesions 1.8 x 1.2 cm;  1.6 x 0.6 cm | liver lesions 1,8 x 1,2 cm;  1.6 x 0.6 cm | liver lesions 1.8 x 1.2 cm;  1.6 x 0.6 cm |  |  | SD |
| 23 | NED | paraaortic 1.1 x 0.9 cm |  |  |  | PD |
| 24 | NED | NED | NED | NED | NED | NE |
| 25 | NED | NED | NED | NED | NED | NE |
| 26 | 2 lesions 3^rd^ thoracic vertebra 19 x 4 mm;  8^th^ thoracic vertebra 17 x 5 mm | 2 lesions 3^rd^ thoracic vertebra 19 x 4 mm;  8^th^ thoracic vertebra 17 x 5 mm | 3 lesions 3^rd^ thoracic vertebra 19 x 4 mm;  8^th^ thoracic vertebra 17 x 5 mm;  NEW: 1^st^ thoracic vertebra /7^th^ cervical vertebra  24 x 11 mm |  |  | PD |
| 27 | NED | NED | NED | new lesion 11^th^ thoracic vertebra 7 mm | new lesion 11^th^ thoracic vertebra 7 mm | PD |
| 28 | pelvic lesion  not measurable | pelvic lesion  not measurable | pelvic lesion  not measurable | ND |  | SD |
| 29 | NED | NED | NED | NED | NED | NE |
| 30 | NED | NED | NED | NED | NED | NE |
| 31 | NED | NED | not done |  |  | NE |
| 32 | NED | NED | NED | NED | NED | NE |
| 33 | 3^rd^ thoracic vertebra 1.2 x 1.6 cm | 3^rd^ thoracic vertebra 1.2 x 1.6 cm | 3^rd^ thoracic vertebra 0.9 cm | 3^rd^ thoracic vertebra 0.9 cm | 3^rd^ thoracic vertebra  non measurable | PR |
| 34 | liver lesions >20  non measurable | liver lesions >20  non measurable | liver lesions >20  non measurable | ND | liver lesions >20  non measurable | SD |
| 35 | soft tissue  os sphenoidale 1.7 cm | soft tissue  os sphenoidale 1,7 cm | soft tissue  os sphenoidale 1,7 cm | soft tissue  os sphenoidale 1.7 cm | soft tissue  os sphenoidale 1.7 cm | SD |
| 36 | adrenal 3.9 x 2.9 x 1.6 cm | adrenal 3.0 x 2.6 x 1.3 cm | adrenal 3.0 x 2.6 x 1.3 cm | adrenal 3.0 x 2.6 x 1.3 cm,  + NEW LESION |  | PR |
| 37 | diffuse meningeal | diffuse meningeal;  NEW intracerebral lesion |  |  |  | PD |
| 38 | retroperitoneal | retroperitoneal | retroperitoneal | retroperitoneal | retroperitoneal | SD |
|  | 3.0 x 2.4 x 1.2 cm | 3.0 x 2.4 x 1.2 cm | 3.0 x 2.4 x 1.2 cm | 3.0 x 2.4 x 1.2 cm | 3.0 x 2.4 x 1.2 cm |  |
|  |  |  |  |  |  |  |
|  |  |  |  |  | CR: | 1 |
|  |  |  |  |  | PR: | 3 |
|  |  |  |  |  | SD: | 9 |
|  |  |  |  |  | Response rate | 4/24 = 17% |
|  |  |  |  |  | CR rate | 1/24 = 19% |

Blue indicates CR and green PR. CNS, central nervous system; CR, complete response; CT, computed tomography; INRC, International Neuroblastoma Response Criteria; le, left; MRI, magentic imaging resonance; NED, no evidence of disease; ND, not determined; NE, not evaluable; PR, partial response; RECIST, Response Evaluation Criteria in Solid Tumours; ri, right; SD, stable disease.
