# Supplementary material for: Long-term, continuous infusion of single-agent dinutuximab beta for relapsed/refractory neuroblastoma: an open-label, single-arm, Phase 2 study
Source: Br J Cancer. 2023 Oct 10;129(11):1780–6. doi: 10.1038/s41416-023-02457-x (PMC10667538; doi:10.1038/s41416-023-02457-x)
Supplement: Supplementary file 6 — Table S5: Response according to INRC components: bone marrow response [file 41416_2023_2457_MOESM6_ESM.docx]

**Table S5: Response according to INRC components: bone marrow response**

| **Patient** | **Baseline** | **Mid evaluation** | **End of treatment** | **12-week FU** | **24-week FU** | **Best response** |
| --- | --- | --- | --- | --- | --- | --- |
| 1 | positive | negative | negative | negative | negative | CR |
| 2 | negative | negative | negative | negative |  | NE |
| 3 | negative | negative | negative |  | negative | NE |
| 4 | negative | negative | negative | negative | negative | NE |
| 5 | positive | negative | negative | negative | negative | CR |
| 6 | negative | negative | negative | negative | negative | NE |
| 7 | negative | negative |  |  |  | NE |
| 8 | positive | negative |  |  |  | CR |
| 9 | positive | less positive |  |  |  | PR |
| 10 | negative | negative | negative | negative |  | NE |
| 11 | positive | negative |  |  |  | CR |
| 12 | negative | negative | negative | negative | negative | NE |
| 13 | negative | negative |  |  |  | NE |
| 14 | negative | negative | negative |  |  | NE |
| 15 | negative | negative | negative | negative | negative | NE |
| 16 | negative | negative | negative |  |  | NE |
| 17 | negative | negative | negative | negative | negative | NE |
| 18 | positive | negative | negative |  |  | CR |
| 19 | negative | negative | negative |  |  | NE |
| 20 | positive | negative | negative |  |  | CR |
| 21 | negative | negative |  |  |  | NE |
| 22 | negative | negative | negative |  |  | NE |
| 23 | positive | positive |  |  |  | SD |
| 24 | negative | negative | negative | negative ? | negative | NE |
| 25 | positive | negative | negative |  |  | CR |
| 26 | positive | negative |  |  |  | CR |
| 27 | positive | negative | negative | positive | negative | CR |
| 28 | negative | negative | negative | positive |  | PD |
| 29 | negative | negative | negative | negative | negative | NE |
| 30 | negative | negative | negative | negative |  | NE |
| 31 | negative | negative | negative |  |  | NE |
| 32 | positive | negative | negative | negative | negative | CR |
| 33 | positive | negative | negative |  |  | CR |
| 34 | negative | negative | negative | negative |  | NE |
| 35 | negative | negative | negative | negative |  | NE |
| 36 | positive | negative | negative |  |  | CR |
| 37 | negative |  |  |  |  | NE |
| 38 | negative |  |  |  | CR: | 12 |
|  | positive | 14 |  |  | PR: | 1 |
|  |  |  |  |  | SD: | 1 |
|  |  |  |  |  | Response rate | 13/14 = 93% |
|  |  |  |  |  | CR rate | 12/14 = 86% |

Blue indicates CR, green PR and red a positive response. CR, complete response; FU, follow-up; INRC, International Neuroblastoma Response Criteria; NE, not evaluable; PD, progressive disease; PR, partial response; SD, stable disease.
